# Effects of meteorological factors on the composition of selected fungal spores in the air

**DOI:** 10.1007/s10453-014-9347-1

**Published:** 2014-09-12

**Authors:** Agnieszka Grinn-Gofroń, Beata Bosiacka

**Affiliations:** Department of Plant Taxonomy and Phytogeography, Faculty of Biology, University of Szczecin, Wąska 13 Street, 71-415 Szczecin, Poland

**Keywords:** Fungal spore composition, Meteorological parameters, CCA

## Abstract

The aim of the study was to determine functional relationships between composition of air spora and meteorological factors, using multivariate statistical technique: canonical correspondence analysis (CCA). Analyses were conducted for the data collected during the 4 year (2007–2010) and, in order to show the dynamics of such relationships, for each year separately. The CCA results indicated that all statistically significant variables accounted for 15.3 % of the total variance in the spore data in the 4 years. The largest amount of the total variance was explained in this period by the mean air temperature (9.2 %). The meteorological factors impacted spore composition differently in different years, when analysis was done for each year separately. The highest values of the total variance in the spore data, explained by the statistically significant variables, were found in 2010 (32.3 %), with the highest contribution of mean air temperature (23.8 %). In that year, the above-mentioned parameter had the lowest value in comparison to other years. Canonical correspondence analysis provides not only a comprehensive assessment of the impact of meteorological factors on specific spore combinations in the air, but also informative graphical presentations of the results, illustrating the correlation between the occurrence of particular spore taxa and meteorological variables.

## Introduction

Fungal spores are an ever-present component of the air with concentrations and compositions known to fluctuate according to the complex interaction between biological and environmental factors such as: geographical location, air pollution, weather conditions, human activity and local source of vegetation. Effects of meteorological factors on the spore occurrence in the air have been highlighted by many authors for many geographical domains (Hasnain 1993; Hjelmroos 1993; Katial et al. 1997; Li and Kendrick 1995a; Oliveira et al. 2009a; Troutt and Levetin 2001). Aerobiological studies enable us to ascertain the concentration of the fungal spores present in the atmosphere and give better understanding of the relationship between their concentrations and the weather parameters. Most of these studies were based on simple descriptive statistics, such as Pearson or Spearman’s correlation coefficients, or on multiple technique, such as the Duncan multiple range test and multiple regression model (Katial et al. 1997; Angulo-Romero et al. 1999; Mitakakis et al. 2001; Troutt and Levetin 2001; Stennett and Beggs 2004). For the three types of spores (*Alternaria*, *Cladosporium* and *Ganoderma*), the predictive models were developed using advanced statistical methods like: artificial neural networks (ANN) and multivariate regression trees (MRT) (Grinn-Gofroń and Strzelczak 2008a, 2008b, 2009, 2011). All these studies put emphasis on the statistical analysis of the correlation between the level of concentration of particular fungal spore types and weather parameters, however do not examine the complex composition of spores and its dependence on meteorological factors. These phenomena have been analyzed in only a few studies (Hjelmroos 1993; Li and Kendrick 1995a, 1995b).

The aim of this study was to determine the dependence between selected meteorological factors and the composition of spores in the air during the 4-year period. In order to show the dynamics of these relationships, the same analyses were performed for each year separately.

## Materials and methods

The data of this study were collected in Szczecin, which is located in the north-western part of Poland, at latitude and longitude of approximately 53°25′N, and 14°35′E, respectively. The climate in Szczecin is temperate with a clear influence of the Baltic Sea. The driest months are February and March; the rainiest and hottest—July; and the coolest—January. The annual mean temperature is around 8 °C, and the difference between warmer and colder monthly averages is significant. Annual air humidity is between 70 and 75 %, and the total annual precipitation varies between 500 and 550 mm (Woś 1999).

Samples were collected using the methods described by the British Aerobiology Federation (1995). The investigation was based on aerobiological monitoring performed in 2007–2010. Airborne fungal spores were continuously monitored, using a seven-day Hirst-type volumetric spore trap manufactured by Lanzoni, Italy, with a flow rate of 10 L min^−1^. The sampler was placed on the roof of University of Szczecin at the height of 21 m above the ground level approximately 5 km from the city center. Sampler drums were changed weekly, and the tapes were cut into 48-mm segments representing the previous 7 days. Spores were trapped onto a Melinex adhesive tape, and cut into daily parts. Each segment of tape was placed on a microscope glass, colored using lacto phenol Cotton Blue mountant and closed with cover slips, and sealed with nail varnish the next day as described in details by the British Aerobiology Federation (1995). The daily mean concentration of the number of fungal spores was determined using an optical microscope at a magnification of 400× along one lengthwise traverse. Thus, the final counts of fungal spores were expressed as average daily number of spores per cubic meter of air. In order to verify the accuracy of calculations performed under the microscope, most of the samples have been reviewed using a microscopic camera connected to a computer screen.

The spore data were analyzed in order to determine the beginning, the end and the duration of a spore season, using the 90 % method. The beginning of a season was defined as the date when 5 % of the seasonal cumulative spore count was trapped and the end of a season as the date when 95 % of the seasonal cumulative spore count was reached.

The meteorological data covering 4 years of studies were provided by the Automatic Weather Station (Vaisala MAWS101). The meteorological station was located in the immediate neighborhood of the Lanzoni trap.

### Data analysis

Effects of selected weather parameters on airborne fungal spore composition were assessed using the software package CANOCO v. 4.5 (ter Braak and Šmilauer 2002). Fungal spore distribution patterns in relation to meteorological variables were determined by canonical correspondence analysis (CCA), after detrended correspondence analysis (DCA) results detected an unimodal structure of the spore data. A complete CCA was performed including eight selected meteorological variables: dew point temperature (DP), precipitation (PRECIP), relative humidity (RH), minimum air temperature (TMIN), maximum air temperature (TMAX), mean air temperature (TME), maximum wind speed (WINDMAX) and mean wind speed (WINDME). The spore and meteorological data were log transformed (by a modified formula available in CANOCO): Y_ki_^*^ = log(A*y*_*ki*_ + B), where *y*_*ki*_ is the concentration of *k* spores in *i* sample; the coefficients A and B are standard set as 1.

Tests of significance of the first and all canonical axes were evaluated for the statistical assessment of the relation between airborne fungal spores and environmental variables (Monte Carlo test: 499 permutations under reduced model). The Monte Carlo permutation test was further applied in order to determine the statistical significance of meteorological variables in explaining the fungal spore variation in the air. For this purpose, “stepwise forward selection” of explanatory variables was used (available in CANOCO). The procedure started with selection of the best explanatory variable (a variable that best explains the total data variance), and the sequence (rank) of other variables was determined according to their decreasing importance in explaining the total variance in the data set, together with the previously selected variables. Therefore, a value of “extra fit” was calculated (Lambda A), which is a change in the sum of all CCA eigenvalues when another variable is added. Additionally, statistical significance of each variable was calculated (*p*). Variation in the airborne fungal spore composition, explained by meteorological variables included in the analysis, was expressed in a percentage—the ratio of the sum of all canonical eigenvalues to the value of total variance (total inertia). Variation in the spore composition explained by individual variables was calculated from the ratio of Lambda A to the total variance (total inertia), expressed in a percentage.

## Results

The DCA results revealed that the gradient length represented by the first ordination axis was greater than 3 SD in all cases (in 2007: 3.168, in 2008: 3.349, in 2009: 4.014, in 2007–2010: 3.708); therefore, the direct CCA ordinations was performed.

The CCA results obtained indicated that all the applied variables accounted for 16.5 % of the total variance in the spore data in 4 years (2007–2010) (Table 1). First axis and all canonical axes were significant as tested by the unrestricted Monte Carlo permutation test (*p* = 0.002) (Table 2).

**Table 1:** Summary of CCA for samples collected in Szczecin (NW Poland)

	Axes	2007	2008	2009	2010	2007–2010
Eigenvalues	I	0.122	0.088	0.088	0.175	0.065
	II	0.015	0.045	0.014	0.010	0.018
	III	0.011	0.005	0.005	0.003	0.003
	IV	0.004	0.004	0.003	0.002	0.001
Fungal spores-environment correlations	I	0.627	0.632	0.655	0.785	0.603
	II	0.381	0.437	0.334	0.355	0.310
	III	0.338	0.361	0.290	0.196	0.181
	IV	0.330	0.331	0.189	0.243	0.120
Cumulative percentage variance of fungal spore data	I	19.1	13.7	14.9	29.8	10.0
	II	21.4	20.6	17.2	31.5	12.8
	III	23.1	21.4	18.2	32.0	13.2
	IV	23.8	22.1	18.6	32.3	13.4
Cumulative percentage variance of fungal spores-environment relationship	I	79.1	61.1	78.6	91.2	74.3
	II	88.5	92.0	91.1	96.4	95.2
	III	95.6	95.5	95.9	97.9	98.1
	IV	98.4	98.4	98.2	99.1	99.3
Sum of all eigenvalues/total inertia		0.639	0.642	0.591	0.589	0.654
Sum of all canonical eigenvalues		0.154	0.144	0.112	0.192	0.108
Percentage of explained fungal spore data variance		24.1	24.4	20.3	32.6	16.5

**Table 2:** Results of the tests of significance of the first and all canonical axes

	Axes	2007	2008	2009	2010	2007–2010
Eigenvalues	I	0.122	0.088	0.088	0.175	0.065
*F*-ratio		35.706	22.551	22.363	73.703	69.328
*p* value		0.0020	0.0020	0.0020	0.0020	0.0020
Trace	I–IV	0.154	0.144	0.112	0.190	0.088
*F*-ratio		6.015	5.131	3.735	10.551	12.133
*p* value		0.0020	0.0020	0.0020	0.0020	0.0020

The results of stepwise forward selection of variables revealed that in the above-mentioned period, four out of eight included meteorological variables (DP, RH, TME and WINDME) were statistically significant (*p* ≤ 0.05), and accounted for 15.3 % of the total variance in the airborne fungal spore occurrence. The largest amount of the total variance was explained by the TME (9.2 %) (Table 3).

**Table 3:** Forward selection results with the test of variable significance for samples collected in Szczecin (NW Poland)

Variables	Lambda A					Explained data variance (%)					*p*				
	2007	2008	2009	2010	2007–2010	2007	2008	2009	2010	2007–2010	2007	2008	2009	2010	2007–2010
DP	**0.09**	**0.08**	**0.01**	**0.02**	**0.02**	**14.1**	**12.5**	**1.7**	**3.4**	**3.1**	**0.002**	**0.002**	**0.004**	**0.002**	**0.002**
RH	**0.01**	**0.04**	0.01	**0.01**	**0.01**	**3.1**	**6.2**	1.7	**1.7**	**1.5**	**0.004**	**0.002**	0.426	**0.005**	**0.002**
TME	**0.02**	0.00	**0.08**	**0.14**	**0.06**	**1.6**	0.0	**13.5**	**23.8**	**9.2**	**0.002**	0.078	**0.002**	**0.002**	**0.002**
TMIN	0.01	0.01	0.01	0.00	0.00	1.6	1.5	1.7	0.0	0.0	0.056	0.216	0.128	0.456	0.128
TMAX	0.01	0.01	0.00	0.00	0.01	1.6	1.5	0.0	0.0	0.0	0.162	0.450	0.924	0.150	0.170
WINDME	**0.01**	0.00	0.00	**0.01**	**0.01**	**1.6**	0.0	0.0	**1.7**	**1.5**	**0.010**	0.332	0.188	**0.002**	**0.030**
WINDMAX	0.00	0.00	0.00	0.00	0.01	0.0	0.0	0.0	0.0	1.5	0.348	0.738	0.846	0.860	0.082
PRECIP	0.00	0.00	0.00	**0.01**	0.00	0.0	0.0	0.0	**1.7**	0.0	0.156	0.620	0.426	**0.048**	0.378

Bold values are statistically significant (*p* ≤ 0.05)

**Fig. 1:**
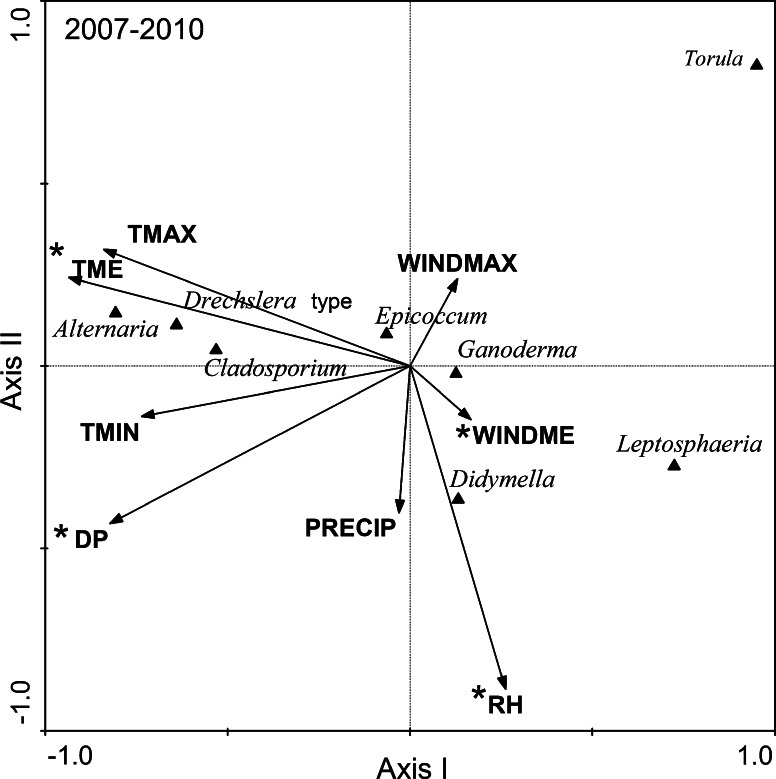
Diagram of fungal spore and meteorological variable ordination along the first two CCA axes for samples collected in Szczecin (NW Poland); total for all years of the study (2007–2010)

According to the ordination diagram (Fig. 1), the occurrence of spores of *Alternaria, Drechslera* type and *Cladosporium* was associated with the highest TME and DP values, *Ganoderma*, *Epicoccum* and *Didymella*—with moderate values, whereas *Leptosphaeria* and *Torula*—with the lowest values. Following the gradient of increasing relative humidity of air, the occurrence of spores of *Didymella* and *Leptosphaeria* was related to the highest RH values, whereas *Torula* and *Alternaria*—to the lowest values. The spores of *Ganoderma*, *Epicoccum, Drechslera* type and *Cladosporium* occurred at moderate values of relative humidity of air. The maximum abundance of *Leptosphaeria* and *Didymella* spores was observed at the highest values of the last statistically significant variable—WINDME, at the moderate values—*Ganoderma*, *Epicoccum* and *Torula*, and at the lowest values—*Cladosporium*, *Drechslera* type and *Alternaria*.

Additionally CCA was performed for each of the 4 years separately in order to determine the dynamics of the relationship between the composition of fungal spores in the air and meteorological variables. In 2007 and 2008, similar values of the total variance in the spore data, explained by the meteorological factors, were found (24.1–24.4 %), slightly lower in 2009 (20.3 %), while the highest—in 2010 (32.6 %) (Table 1).

In 2007, there were four statistically significant variables: DP, RH, TME and WINDME, which explained a total of 20.4 % of the variance of the spore composition. The largest contribution to the total variation of fungal spore composition in that year had DP (14.1 %) (Table 3). In that year, the annual mean of dew point temperature was the highest in comparison with other years (Table 4).

**Table 4:** Annual, mean values of meteorological parameters for Szczecin in 2007–2010

Meteorological parameters	2007	2008	2009	2010
Annual mean of air temperature (°C)	9.6	9.8	9.5	9.0
Annual mean of dew point temperature (°C)	6.4	5.2	5.4	5.0
Annual sum of precipitation (mm)	788.2	669.9	644.4	807.7
Annual mean of relative humidity (%)	81.8	79.5	81.7	81.5
Annual mean of wind speed (m/s)	3.4	2.9	3.1	5.0

In 2008, a statistically significant variables were DP and RH, which explained 18.7 % of the total variance. The largest range of total variation in the spore composition was explained by the DP (12.5 %), as in the previous year. Range of total variation, which was explained by RH (6.2 %), was the highest in comparison to the variability of the spore composition, which was explained by this variable in other years (Table 3). In that year, the annual mean of relative humidity was the lowest in comparison to other years (Table 4).

In 2009, statistically significant were two meteorological variables: DP and TME, which explained a total of 15.2 % of the variance. The largest range of the total variance in the spore data was explained by the TME (13.5 %), as in the next year (Table 3).

In 2010, as many as five variables were statistically significant: DP, RH, TME, WINDME and PRECIP, that explained a total of 32.3 % of the variance of the spore composition. The largest contribution to the total variation of fungal spore composition in that year was TME (23.8 %) (Table 3). In that year, the annual mean of air temperature was the lowest in comparison with other years. The annual mean of dew point temperature also reached the lowest value, compared with other years, and the annual sum of precipitation and annual mean of wind speed reached the highest values (Table 4).

Only one meteorological variable (DP) had a statistically significant impact on the total variance of the occurrence of airborne fungal spores in all 4 years (explaining from 1.7 to 14.1 % of the total variance). The other statistically significant variables impacted spore composition differently in different years: RH explained a part of the total variance in the occurrence of spores in 2007, 2008 and 2010 (from 1.5 to 6.2 %), TME—in 2007, 2009 and 2010 (from 1.6 to 23.8 %), WINDME—in 2007 and 2010 (from 1.5 to 1.7 %), PRECIP—only in 2010 (1.7 %) (Table 3).

**Fig. 2:**
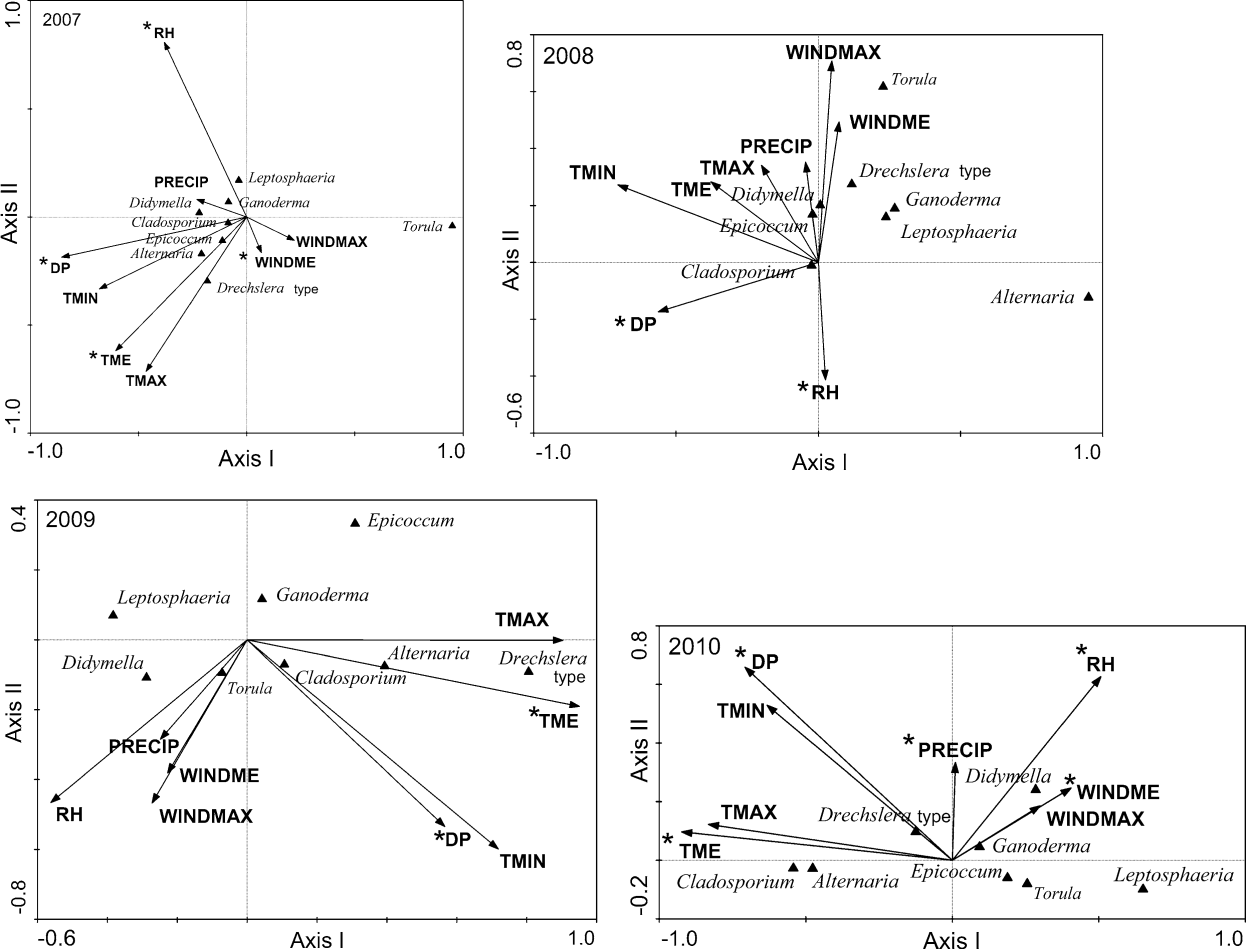
Diagrams of fungal spore and meteorological variable ordination along the first two CCA axes for samples collected in Szczecin (NW Poland); separately for each year of the study (2007, 2008, 2009, 2010)

According to the ordination diagrams, made separately for each year of the study (Fig. 2), and taking into account only statistically significant meteorological variables, in 2007, almost all examined airborne fungal spores (with the exception of *Torula*) occurred in the same, moderate range of values of meteorological variables. In 2008, most of spore types occurred also at moderate values of meteorological variables, but with two exceptions (*Torula* and *Alternaria*). In 2009, greater differentiation was observed in the occurrence of spore types. In 2010, the presence of most spore types was related to the lowest values of meteorological variables.

## Discussion

The fungal spore concentrations in the air have been extensively studied in modern times. It is a matter of prime concern to the mycologist studying the epidemiology of plant diseases. In medicine, the importance of fungal spores in the air has been emphasized in connection with allergic reactions with a wide spectrum of clinical forms. Because fungal spores are an important component of bioaerosol and also considered to act as an indicator of the level of atmospheric bio-pollution, a better understanding of these phenomena demands a detailed survey of airborne particles. In addition, many types of spores are allergens, and their presence in the air in suitable formulations is very important from the human health point of view (Gravesen 1979).

Meteorological factors are known to influence the production, release and dispersal of aeroallergens, which vary from place to place and from season to season. In this study, results of CCA from the 4-year period showed four meteorological variables (mean air temperature, dew point, relative humidity and mean wind speed), which most influenced the composition of spores in the air.

The largest range of variation in spore composition in the air in north-western Poland (2007–2010) was explained by mean air temperature. As have been obtained in other studies, the occurrence of *Cladosporium, Alternaria, Drechslera* type, *Ganoderma* and *Epicoccum* spores is positively correlated with the air temperature (Hjelmroos 1993; Li and Kendrick 1995a; Herrero et al. 1996; Craig and Levetin 2000; Troutt and Levetin 2001; Peternel et al. 2004; Kasprzyk et al. 2004; Stępalska and Wołek 2005; Grinn-Gofroń 2008; Oliveira et al. 2009b). Such composition of spores present in high concentrations in the air is one of the main causes of allergies in humans (Gravesen 1979; Chapman and Williams 1984; Mitakakis and Guest 2001; Oliveira et al. 2007). It occurs most frequently in summer and early autumn. Airborne fungi (especially allergenic types) are mainly mesophilic (optimal temperature for growth 20–40 °C), and some are psychrotolerant (optimum below 20 °C) (Gravesen 1979).

Among the above-mentioned spore types, in our 4-year study, the maximum abundance of *Alternaria*, *Drechslera* type and *Cladosporium* spores was observed at higher mean temperature values than those of *Ganoderma* and *Epicoccum* spores.

Among other types of spores in our study, the occurrence of *Didymella* spores was associated with the moderate mean temperature values, nevertheless the relationship was weaker than in case of previous spore types. Wahl and Kersten (1991) found that the release and dispersal of *Didymella* spores was not greatly affected by air temperature.

*Leptosphaeria* and *Torula* spores were related to the lowest mean temperature values in north-western Poland; nonetheless, in case of *Torula* spores, the relationship was very weak. *Leptosphaeria* was reported as potentially allergenic in Great Britain, due to the fact that a few percent of vulnerable patients reported positive responses in skin tests (Lacey 1996). Hasnain (1993) reported that number of *Leptosphaeria* spores had a statistically significant correlation with the air temperature in the night.

According to the reports of Chapman and Williams (1984), *Torula* can be a potential allergen, which in some sensitive people evokes hay fever and asthma symptoms. *Torula* spores occur frequently, however, in low numbers and represent only a few percent of the aeromycoflora tested (Ibáńez Henriquez et al. 2001; La-Serna et al. 2002; Oliveira et al. 2007; Sen and Asan 2001; Vittal and Krishnamoorthi 1981). Probably due to the occurrence in the air in low concentrations, *Torula* does not have a strong dependence with meteorological parameters.

The second most important meteorological parameter which affected the composition of spores in the air in our study was dew point temperature. This factor is strongly associated with the previously discussed meteorological variable, and that results in similar relationships. The maximum abundance of *Alternaria* spores was observed at the highest values of dew point temperature; however, it was not strong as mean air temperature. Sensitivity analysis of the artificial neural network showed dew point temperatures as the variable positively influencing the presence of *Alternaria* (Grinn-Gofroń and Strzelczak 2008a). The contrary results for dew point temperature were obtained by Troutt and Levetin (2001). Differences in the results from the discussed studies are probably caused by various methodologies applied. In this investigation, the long-term time series of daily concentrations were analyzed while Grinn-Gofroń and Strzelczak (2008a) created artificial neural network model for daily concentrations in spore seasons. In turn, multiple regression models by Troutt and Levetin (2001) concerned daily concentrations only in May 1999 and that month represented climatic extremes (unusually high precipitation). Moreover, artificial neural networks, opposite to multiple regression, can model nonlinear relationships. Grinn-Gofroń and Strzelczak (2008a) revealed that the dependence of *Alternaria* spore concentration on dew point temperature and humidity was unimodal with a decline in spore content at high values of the discussed meteorological parameters. High precipitation in May 1999 studied by Troutt and Levetin (2001) caused high humidity and dew point temperature. Thus, the situation might have corresponded with meteorological conditions after the inflection point of *Alternaria*—dew point/humidity curves.

The third most important meteorological variable in our 4-year study was relative humidity. It is certain that relative humidity has an important influence on the aeromycoflora, mainly on spores belonging to Ascomycetes and Basidiomycetes. Inglod (1971) wrote that ascus dehiscence mechanisms are basically hydrostatic in nature. In many Ascomycetes, high osmotic pressure, which is elevated by transformation of osmotically inactive carbohydrates into osmotically active sugars, develops within the ascus either by the direct absorption of water from the air or by the swelling of muscilage within the ascus (Moore-Landecker 1990). The resulting pressure leads to an explosive release of the ascospores into the turbulent atmosphere. Ascospores are often abundant in the air during and after rainfall, when the level of humidity is sufficiently high (Allitt 1986).

Active discharge of basidiospores in most species of Basidiomycota is powered by the rapid movement of a droplet of fluid, called Buller’s drop, over the spore surface. Upon maturity of a basidiospore, carbohydrates present in the cell wall begin to serve as condensation loci for water vapor in the air. At the pointed tip of the spore closest to the supporting basidium, Buller’s drop accumulates as a large, spherical water droplet. At the same time, condensation occurs in thin film on the adaxial face of the spore. When these two bodies of water coalesce, the release of surface tension and the sudden change in the center of mass leads to sudden discharge of the basidiospore (Van Neil et al. 1972; Webster et al. 1984; Noblin et al. 2009).

Spores, which the highest levels recorded during the dry and hot weather (*Alternaria, Torula, Cladosporium, Drechslera* type, *Epicoccum*) in case of humidity, have shown an inverse dependence. The concentrations of these “dry-weather spore types” decreased. Sen and Asan (2001), Stępalska and Wołek (2005), Grinn-Gofroń (2008), Oliveira et al. (2009b) found a negative correlation between concentration of *Cladosporium, Alternaria, Drechslera* type, *Epicoccum* spores and relative humidity. In our study, the maximum occurrence of *Alternaria* spores was also associated with the lowest relative humidity, while the maximum occurrence of spores of *Didymella* and *Leptosphaeria* was related to the high humidity. Similar results have been obtained in other studies (Harries et al. 1985; Richardson 1996). Richardson (1996) concluded that the high humidity resulting from low temperature and dew formation in the early hours was the reason for high concentrations of *Didymella* spores in the atmosphere.

The active (violent or forcible) and passive discharge of basidiospores occur in almost equal proportions in the Basidiomycota. Discharge of basidiospores requires atmospheric moisture, although the mechanism is not completely understood. Basidiomycetes exhibiting a forcible mechanism of discharge probably use an interaction between a gas-bubble mechanism and an electrostatic mechanism (Saville 1965). If a typical gilled Hymenomycetes can absorb adequate moisture from a rain, it likely can maintain a stable microclimate inside the basidiocarp to enhance spore release and dispersal regardless of the humidity surrounding the basidiocarp. Spore release would continue until the moisture available in the basidiocarp, and outlying mycelium was used up (Dube 2005). In our study, the maximum abundance of *Ganoderma* spores was observed at the moderate values of relative humidity, although—as was stated by Haard and Kramer (1970)—basidiospores are frequently seen when the air humidity is high. *Ganoderma* spores are considered to be a moist-air spore type because their concentrations showed marked seasonal and diurnal differences with the highest numbers during the wet season, and water is an important factor involved in its spore release. This agrees with the reports of McCracken (1987) and Hasnain et al. (2004), who stated that humidity levels of about 70 % were associated with increased concentrations of *Ganoderma* spores. The diurnal pattern with a peak early in the morning and the secondary peak late in the evening was noted in England (London and Worcester) (Lacey 1962; Sadyś et al. 2014), Canada (Tarlo et al. 1979) and New Zealand (Hasnain et al. 1984).

The last statistically significant variable in our 4-year study was wind speed. This factor plays an important role in spore release and dispersal. McCartney (1991) noted that information on the strength of spore attachment and the values of threshold wind speed are not known for the vast majority of fungi. The long-distance dispersal of spores depends on wind conditions, but the detachment of spores dispersed in dry conditions is also strongly influenced by wind (Mallaiah and Rao 1982). The studies of Lin and Li (2000) showed a strong negative correlation between fungal spore concentration and wind speed when the wind speed was under 5 m/s. However, the fungal concentration increased as the wind speed was higher than 5 m/s. The authors took this to be evidence of the dilution effect of wind speed being overcome by more particles being raised in higher winds. Jones and Harrison (2004) concluded that maximum wind speed has to exceed a threshold speed to remove material from a surface by either blow or movement of the surface. However, at higher speed, spore concentrations may become diluted. Packe and Ayers (1986) suggest that turbulent winds could increase the release of fungal spores or draw up sedimented fungal spores and resuspend them in the air.

In our analysis *Alternaria*, *Cladosporium* and *Drechslera* type spores showed dependence on the lowest value of mean wind speed, whereas *Leptosphaeria* and *Didymella*—on the highest. A negative correlation between the *Alternaria* spores count and the wind speed was observed by Levetin and Dorsey (2006) and by Hasnain (1993). Lopez and Salvaggio (1983) also confirmed that high wind speed produced a decreased atmospheric concentration of *Alternaria* spores count. The lack of correlation between the concentration of *Cladosporium* spores and wind speed was recorded by Levetin and Dorsey (2006), Hasnain (1993), Lopez and Salvaggio (1983), and the same results for *Drechslera* type spore concentration were recorded by Troutt and Levetin (2001).

The effects of meteorological factors varied among years. In our analysis, similarly to Li and Kendrick (1995b), the mean values of meteorological parameters were generally more important than their maximum and minimum. Differences in the dependences between meteorological parameters and fungal spore levels can be observed in every year, and this is most likely due to the different average values of meteorological parameters in every year studied. However, the spore behavior is a dynamic and complex phenomenon, and meteorological factors alone do not reflect the global status of the atmosphere. Therefore, it is difficult to separate the individual effects of different meteorological parameters, since fungi react simultaneously to a combination of factors (Azcón-Bieito and Talón 2000). All methods used in the aerobiological analysis should be selected to describe the effects of all meteorological factors on the concentration and composition of spores in the air. Such results, related to the ecology of fungi, would give more reliable view of fungal spore occurrence in the air.
